# Large Aerodigestive Tract Foreign Body Extraction Complicated by End-Stage Dementia

**DOI:** 10.7759/cureus.12822

**Published:** 2021-01-20

**Authors:** Dharam Persaud-Sharma, Sayoni Saha, Jacob Poynter, Deepa Danan, Nicholas Nedeff, Jack Hagan

**Affiliations:** 1 Department of Anesthesiology, University of Florida, Gainesville, USA; 2 Department of Otolaryngology, University of Florida, Gainesville, USA; 3 Department of Anesthesiology, Kendall Regional Medical Center, Miami, USA; 4 Department of Anesthesiology, University of Central Florida, Miami, USA

**Keywords:** foreign object extraction, blocked airway, dementia, anesthesiology, ent, otolaryngology, oral fixation

## Abstract

A foreign body in the upper aerodigestive tract can be life-threatening. It is especially challenging for surgeons and anesthesiologists working in a limited shared workspace. A case is presented of an 83-year-old woman with end-stage dementia afflicted with oral fixation as defined as overeating or putting objects in the mouth other than food. She appeared asymptomatic although she had altered baseline mentation and was found to have ingested a large foreign object. This case provides an opportunity to discuss the unique challenges of performing anesthesia on patients undergoing the extraction of a large upper aerodigestive tract foreign body, complicated by end-stage dementia.

## Introduction

Frontotemporal lobar dementia (FTLD) is an umbrella term that includes several disorders that result in marked deterioration of cognitive and social behaviors [1]. Specifically, patients with FTLD develop hyperorality which can lead to binge-eating, as well as the ingestion of inedible objects [2]. FTLD is the result of selective atrophy of the frontal and temporal lobes with a marked accumulation of tau positive and ubiquitin-positive inclusions found in the brain post-mortem; however, the exact etiology is not clear [2,3]. In later stages of FTLD, many of the patients become non-verbal making hyperorality tendencies an even greater risk secondary to their inability to verbalize or orate the appropriate distress signals of asphyxiation. Additionally, dysphagia may be a progressive feature, result, or complication of dementia. This further complicates the ability to clearly detect changes in mentation and clinical presentation due to an altered baseline mental status with dementia. However, due to the expected difficulties with dysphagia and oral fixation to arise secondary to dementia, ingestion of foreign objects should always remain on the differential diagnoses for any upper airway distress or acute changes in eating behavior. These clinical features are particularly distressing to caretakers because it essentially requires continuous observation of patients to avoid fatal circumstances.

Removing objects from the upper aerodigestive tract can be challenging depending on the object size, geometry, composition, location, method of extraction, and acuity of the patient. Extraction methods vary widely and depend on numerous factors including location, accessibility, and patient tolerance. Foreign bodies of the nasal cavity, oral cavity, and oropharynx can often be removed with direct visualization using forceps, curved hooks, and suction catheters without the need for anesthesia or sedation, except in pediatric patients or in patients with dementia. Objects not easily visualized on an examination can be localized with a combination of history, physical examination, radiographic studies, and flexible nasopharyngolaryngoscopy. Once localized, endoscopic removal is the treatment of choice [4]. Different methods of extraction require varying anesthetic techniques and therefore a coordinated approach between the anesthesiology and surgical team is required. This approach should be individualized based on the acuity of the patient, location, and characteristics of the object, as well as provider preference.

## Case presentation

An 83-year-old woman with advanced frontal lobe dementia, hypertension, and dyslipidemia presented to the emergency department from home due to increased somnolence reported by the patient’s daughter. Given the patient’s general malaise and decreased responsiveness with unknown baseline behavior, the patient was admitted for further evaluation. Her family reported that other than the lack of responsiveness, she was at her baseline level of function. Upon admission, the patient was found to be mostly non-verbal and had a decreased appetite. The daughter, who stayed with the patient at her bedside for the entire duration of the hospitalization, reported that the patient was having a productive cough with increased shortness of breath. On clinical examination, the patient was a thin, elderly woman with muscle wasting of the extremities. She was in a constant lethargic state, non-verbal, arousable to pain, but did not respond to verbal commands. Her vital signs were within normal limits, including oxygen saturation of 95% on room air. Laboratory values revealed hypokalemia and pancytopenia. An incidental 1.6 cm nodular lesion in her thoracic wall and multiple nodules in the thyroid were revealed by chest CT. Saliva specimens were tested for infection and she was found to have a mycoplasma infection. She was treated with antibiotics and inhaled steroid treatment following a pulmonology consultation. Her medical history was otherwise unremarkable. The conclusion by the initial medical team attributed her altered mental status to late-stage dementia.

**Figure 1:**
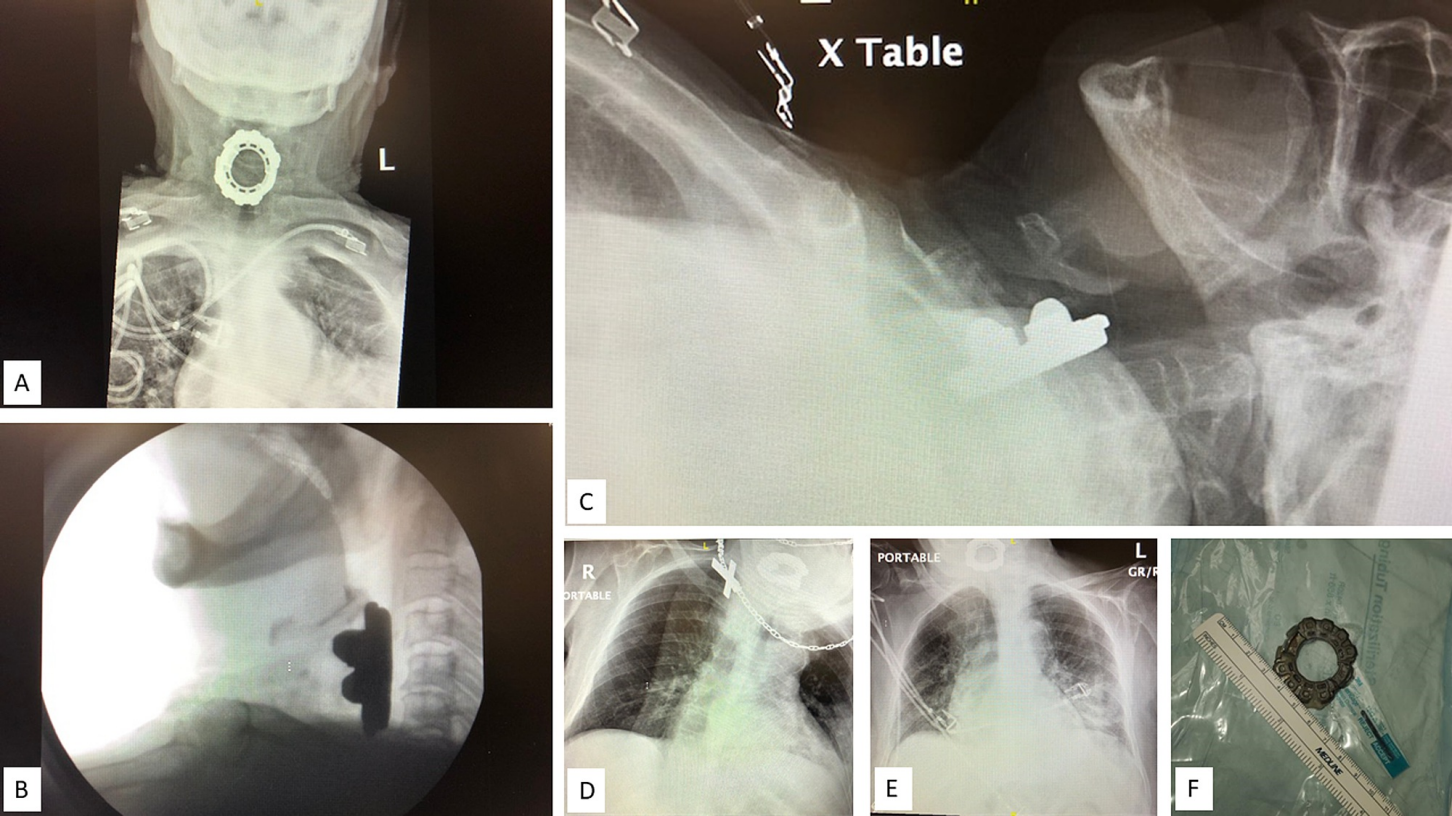
Comparative Chest and Neck Radiographs With Foreign Body in Place vs Extracted (A) Anteroposterior chest radiograph; (B) upright lateral neck radiograph; (C) supine lateral neck radiograph; (D) wide angle anteroposterior neck radiograph; (E) wide angle anteroposterior neck radiograph; (F) 3 cm diameter foreign body extracted (courtesy of Dr. David Gerth).

 A second medical team reviewed the case after a Monday 0630 hrs handoff, three days post-admission. Upon clinical evaluation by the new medical team, the patient was noted to have upper respiratory stridor. A closer re-examination of the previously obtained radiographic images, which included a CT of the brain and chest and chest radiographs, revealed what appeared to be a large foreign body in the hypopharynx-upper esophagus which was previously not described in the radiologist’s report initially read by junior radiology residents. Further investigation included a failed bedside swallow study, and radiographs of the neck soft-tissue and anterior-posterior cervical spine. These revealed a large foreign body in the hypopharynx anterior to the C3-C6 vertebrae measuring 5.4 cm x 2.3 cm x 4.3 cm as seen in Figure 1. The patient was clinically stable, and an ear, nose, and throat (ENT) surgeon was consulted for removal of the foreign object under general anesthesia to avoid erratic movements which could lead to perforation of the esophagus or airway during the extraction process. The object was a "beyblade" children’s toy removed from the hypopharynx by an otorhinolaryngologist surgeon using direct laryngoscopy and Magill forceps while the patient was under general anesthesia with endotracheal intubation using rapid sequence induction without application of cricoid pressure due to the positioning of the foreign object. After removal of the object granulation tissue was found in the post-cricoid region and an area of necrosis was in the left lateral hypopharyngeal wall without perforation. Post-operatively, the patient was more awake and active with a return of appetite. Mental status was unchanged after the removal of the foreign object, but it was noted that her neuropathological oral fixation tendencies dramatically increased.

## Discussion

Foreign body aspiration can be lethal/injurious for any patient but can be even more complicated in the setting of dementia. Patients with dementia are at increased risk for aspiration in the setting of age-related impaired swallow and cough [5]. As discussed previously, objects in the nasal cavity, oral cavity, and oropharynx can often be removed without sedation, however, retrieval without the use of anesthesia requires cooperation from the patient and may not be appropriate in a patient with dementia who has difficulty following directions at baseline [6]. In patients with aspirated items in the glottis, supraglottic or hypopharyngeal regions, the item may be retrieved using a variety of surgical instruments, such as Magill forceps under direct or video laryngoscopy as used in this patient [6]. Endotracheal intubation is typically utilized to protect the airway. Bronchoscopy remains the cornerstone for the diagnosis and management of foreign bodies in the tracheobronchial tree. In the experienced hands, flexible bronchoscopy can be successful in nearly 90% of cases of foreign body aspiration and therefore has become the most used approach in the United States [6]. Flexible bronchoscopy can be performed under local anesthesia and sedation, whereas rigid bronchoscopy requires general anesthesia. However, rigid bronchoscopy offers the advantage of better airway control, better optics, and access to larger instruments [6]. Objects without the complete obstruction of the esophagus, are best removed by a therapeutic esophagogastroduodenoscopy (EGD) within a 24 hour period [7]. A diagnostic algorithm utilized in many radiological training programs for educational and consulting services suggests cheaper less invasive methods first and more invasive and expensive procedures as complexity permits. 

In acute situations where the airway is completely obstructed, oxygenation and securement of the airway are critical. Bag mask ventilation or placement of an endotracheal tube (ETT) should be considered in this setting, incorporating the current guidelines for cardiopulmonary resuscitation and emergency cardiovascular care. An ETT provides a stable airway which can be critical in patients with dementia who may otherwise not be able to protect their airway [8,9]. Sosyal et al. retrospectively reviewed 130 cases of foreign body aspiration and concluded that an ETT can be lifesaving in patients who are cyanotic, somnolent, or in acute respiratory distress [10]. In chronic presentations, the anesthetic and surgical approach should be individualized to the patient, location, size, and composition of the object. Historically, spontaneous ventilation has been thought to be safer to prevent the theoretical risk of controlled or jet ventilation forcing the foreign body distally creating a total obstruction [11]. However, recent studies performed mainly on children have not supported this conclusion [12]. Often, laryngeal foreign bodies can be extracted during laryngoscopy without tracheal intubation. Once removed, the patient can be awoken without intubation depending on the degree of airway edema. If the patient requires airway protection after induction, this can often be performed during laryngoscopy and intubation can be performed around the foreign body or one can attempt to push the foreign body distally into one of the mainstem bronchi where it will not cause bilateral obstruction [6]. If the foreign body is causing asphyxiation and cannot be immediately extracted, providers should be prepared to perform emergent tracheostomy or cricothyroidotomy [6,12,13].

## Conclusions

Patients with dementia are at increased risk for foreign body aspiration. This case report discusses an 83-year-old non-verbal female with frontotemporal dementia who presented to the ED after being found somnolent at home. On further evaluation, she was found to have ingested a large foreign object trapped in the hypopharynx. This foreign body was extracted under general anesthesia with endotracheal intubation using direct laryngoscopy with forceps, a common approach to removing objects from the upper airway. Foreign body aspiration in patients with dementia poses unique challenges given the difficulty of obtaining an accurate history, likely intolerance of object extraction without sedation, and high risk for development of postoperative delirium. Different methods of object retrieval vary widely and ultimately the characteristics of the aspirated object, the degree of obstruction and the patient’s ability to protect their airway should be considered when choosing the best approach for patients with dementia.
